# Validation of a diode‐based phantom for high temporal and spatial measurements in a 1.5 T MR‐linac

**DOI:** 10.1002/acm2.14604

**Published:** 2024-12-25

**Authors:** Stijn Oolbekkink, Jochem W. H. Wolthaus, Bram van Asselen, Madelon van den Dobbelsteen, Bas W. Raaymakers

**Affiliations:** ^1^ Department of Radiotherapy University Medical Center Utrecht Utrecht Netherlands

**Keywords:** Delta4, MR‐linac, quality assurance, time‐resolved dosimetry

## Abstract

**Background:**

For the development and validation of dynamic treatment modalities and processes on the MR‐linac, independent measurements should be performed that validate dose delivery and linac behavior at a high temporal resolution. To achieve this, a detector with both high temporal and spatial resolution is necessary.

**Purpose:**

This study investigates the suitability of a Delta4 Phantom+ MR (Delta4) detector array for time‐resolved dosimetry in the 1.5 T MR‐linac and characterizes the Delta4's performance with dynamic dose delivery, such as beam gating and field modulation during radiation.

**Methods:**

A Delta4 detector was used, including software for time‐resolved dosimetry. First validation experiments were performed and compared to reference measurements. Subsequently, demonstrator measurements were performed to show use cases of the Delta4's time‐resolved dose readouts. An example of such an experiment is the determination of the field speed during a sliding window experiment, traveling between 0.7 and 6.8 cm/s in the cranial‐caudal direction.

**Results:**

Validation experiments of the dose reproducibility and dose rate dependency showed no difference relative to the standard static delivery. The field speed measured by the Delta4 showed an average field speed difference of −0.3% relative to MR‐linac log files. The Delta4 was capable of measuring the dose with high accuracy and temporal resolution during dynamic radiation delivery.

**Conclusion:**

The Delta4 can be used for time‐resolved dosimetry in a 1.5 T MR‐linac.

## 1 INTRODUCTION

MRI‐guided radiotherapy aims to see the tumor and its surrounding healthy tissue before beam delivery and to adapt the treatment plan accordingly.^1^, ^2^, ^3^, ^4^, ^5^ MRI scanning makes it even possible to track the anatomical changes during dose delivery. These MR scans can be used during treatment delivery, in applications ranging from gating to ultimately real‐time full‐replanning.^6^, ^7^

As intra‐fraction adaptations with the patient in the treatment position become more routine, treatment delivery and the quality assurance (QA) become complex. This complexity will be further increased for techniques currently being researched on the MR‐linac, such as MLC‐tracking, in which the MLC positions change based on MR images acquired during dose delivery.^8^

For the development and validation of these dynamic treatment techniques, independent measurements should be performed that validate, for example, the dose output. Traditionally, QA is performed using the cumulative dose of the entire treatment.^9^ However, for dynamic deliveries, it also becomes desirable, and maybe even necessary, to verify the dose delivery and linac behavior at a high temporal resolution. Measuring the time‐resolved dose of a treatment plan allows for the identification of dose deposition modeling errors or delivery errors (e.g., delays, MLC positions, and segment doses) that may not be evident when solely comparing the cumulative dose to the planned dose. To achieve this, a detector with both high temporal and spatial resolutions is necessary.

Detectors, such as ionization chambers, diodes, or scintillation detectors, combined with high‐sampling digitizers, could be used for time‐resolved dosimetry.^10^, ^11^, ^12^, ^13^, ^14^, ^15^ An example of a diode detector is the Delta4 Phantom+ MR (Delta4), manufactured by ScandiDos AB (Uppsala, Sweden), with two orthogonal diode arrays, providing a quasi 3D distribution.^16^ This phantom is currently used for plan QA by measuring the cumulative dose. However, this diode array can also be used for time‐resolved dosimetry.

This study aims to assess the performance of a Delta4 detector for time‐resolved dosimetry in a 1.5 T MR‐linac during dynamic dose delivery.

## 2 MATERIALS AND METHODS

Two measurement sets were performed and analyzed: validation experiments and demonstrator measurements, the latter showcasing the use of time‐resolved dosimetry. First, validation measurements with the time‐resolved software were performed and compared to either published data or cumulative reference measurements. These validation experiments were conducted to evaluate the dosimetric accuracy of the time‐resolved software. Dose reproducibility and dose rate stability were initially compared. Next, the influence of the integration time on the measurement uncertainty of the Delta4 and the treatment record file (TRF) was assessed. Finally, the Delta4's spatial and temporal characteristics were evaluated by assessing various field speeds during a sliding window test.

To demonstrate the use of time‐resolved Delta4 dose readouts, two example experiments were performed. The first experiment measured field positions as a function of time during a sliding window. The second experiment focused on time‐resolved dosimetry for a gated IMRT plan and was compared to the non‐gated reference and planned dose. During both demonstrator experiments, the time‐resolved dose was also calculated based on the TRF and compared to the measurement.

### 2.1 Setup

**FIGURE 1:**
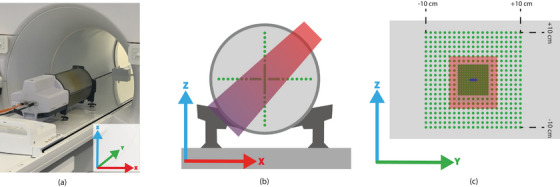
(a) The measurement setup of the Delta4 in the MR‐linac. (b) A schematic transversal and (c) sagittal overview, of measurement setup used for dose reproducibility and dose rate dependency experiments. (c) Shows the three central *y*‐axis diodes (in blue) used for various experiments

All measurements were performed on the 1.5 T Unity MR‐linac (Elekta AB, Stockholm, Sweden), using a Delta4 phantom in combination with dedicated software for time‐resolved dose readouts (see, Figure 1a). The linac was calibrated to deliver 70.1 cGy per 100 MU at isocenter for a 10 × 10 ${\mathrm{cm}}^{2}$ field with 10 cm of buildup, from a gantry angle 90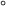 and an SAD of 143.5 cm. Throughout this paper, the IEC 61217 treatment coordinate system was used.^17^ Since the clinical 1.5 T MR‐linac did not support dynamic treatments with moving MLCs during beam‐on, a research version of the MR‐linac control system was used.

The Delta4 is a cylindrically‐shaped PMMA phantom containing two crossed orthogonal detector boards (200 × 200 ${\mathrm{mm}}^{2}$ each) in the sagittal (*zy*) plane and coronal (*xy*) plane, which contains 1069 identical *p*‐type Si‐diode detectors, with a surface area of 0.78 ${\mathrm{mm}}^{2}$. Developed for 1.5 T MR‐linac measurements, the Delta4 features high‐resolution (central region, 60 x 60 ${\mathrm{mm}}^{2}$) and low‐resolution (outer region) detector areas with 5 and 10 mm diode spacing, respectively. The time‐resolved research version of the Delta4 software uses a sampling time of 25 ms for the dose readout. Positioning of the Delta4 was performed using lasers present in the treatment room and phantom markers, after which a calibration plan (Monaco TPS, v5.51.10, Elekta AB, Stockholm, Sweden) was delivered before the measurement series. This plan consisted of four 10 × 10 ${\mathrm{cm}}^{2}$ fields at gantry angles of 225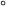, 315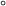, 45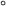, and 135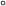, each with 50 MU. Minor positional errors were corrected in the Delta4 software via automated in silico alignment with the calibration plan. Daily dosimetric deviations were corrected using the calibration plan derived correction factor which accounts for day‐to‐day deviations.

#### 2.1.1 Time‐resolved TRF‐based dose calculations

Time‐resolved dose calculations were performed based on parameters stored in the TRF. The TRF sample time is 40 ms and contains the projected MLC positions at the isocenter plane, delivered MU, dose rate, gantry angle, and time‐tag. Time‐resolved 3D dose calculations were performed using the Monte Carlo dose calculation engine of Monaco (GPUMCD, Elekta AB, Stockholm, Sweden) with a clinically validated beam model.^18^, ^19^ This dose engine includes the 1.5 T magnetic field and the effects it has on dose deposition. The TRF‐based dose calculations were performed over each sample of 40 ms found in the TRF log file. Each dose calculation was performed in 3D, simulating the dose delivery in the Delta4, using a 2 × 2 × 2 ${\mathrm{mm}}^{3}$ voxel size with and a 8% statistical uncertainty. Comparisons between Delta4 measurements and TRF‐based dose calculations were conducted in Delta4 diode system coordinates, employing trilinear interpolation on the TRF‐based dose grid to match detector positions. Alignment of the temporal axis between the Delta4 and the TRF file was performed at the first non‐zero dose measurement time point.

### 2.2 Validation experiments

#### 2.2.1 Reference measurements: Dose reproducibility and dose rate dependency

De Vries et al.^16^ found that the dose reproducibility and dose rate stability of the clinical Delta4 software was ≤1%. These were re‐assessed for the research software capable of time‐resolved dose measurements using a field size of 10 × 10 ${\mathrm{cm}}^{2}$, delivered from gantry angle 45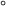 (Figure 1b). The short term dose reproducibility of the Delta4 was assessed by measuring 10 beams of 100 MU, and determining the largest dose deviation from the average measured dose.

The dose rate influence on the Delta4's dose measurement was investigated with a 200 MU beam delivered at six different constant dose rates: 52, 264, 422, 439, 476, and 527 MU/min. The accumulated dose was compared to the measurement with the clinically used dose rate of 439 MU/min. Literature indicates that the dose output variation from a changing constant dose rate of the linac is ≤ 0.1%, making the influence of the linac negligible.^20^

For both the dose reproducibility and the dose rate stability measurements the three centrally located diodes (see, Figure 1c) were used to determine the average dose and standard deviations (SD).

#### 2.2.2 Measurement uncertainty due to integration time

The effect of integration time on the measurement accuracy with which a device can measure dose is an important parameter that needs to be quantified. This quantification, here defined as the measurement uncertainty, represents the minimum quantity of time samples required to achieve confidence in the measured data. The measurement uncertainty of both the Delta4 and TRF‐based time‐resolved dose readouts was assessed as a function of the integration time (number of binned time samples). A beam of 400 MU was delivered to the Delta4 at a 45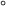 gantry angle with a 10 × 10 ${\mathrm{cm}}^{2}$ field size. The three centrally located diodes (see, Figure 1c) were used to determine the measurement uncertainty. The magnitude of the SD relative to the the average dose, the measurement uncertainty on the dose, of all binned time samples was determined for integration times ranging from 1 (25 ms for Delta4, 40 ms for TRF) to 100 samples (2500 ms for Delta4, 4000 ms for TRF), using a moving summation. This process allowed the identification of the integration interval required to achieve measurement uncertainty of ≤ 1%. This integration interval was subsequently used for the time‐resolved dose assessment of the sliding window experiment (Section 2.3.1).

#### 2.2.3 Spatiotemporal resolution assessment

**FIGURE 2:**
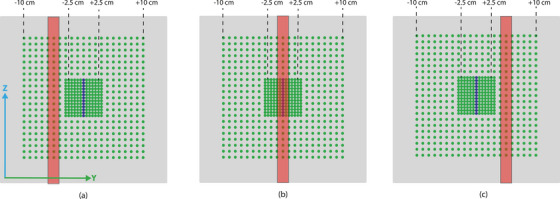
Schematic overview of the movement of the field at three different stages: (a) the utmost −*y* position (−5 cm), (b) the central position (0 cm), and (c) the utmost +*y* position (+5 cm). The diodes in the high resolution region were averaged in the central *z*‐axis (example in blue). In the range of −2.5 cm to +2.5 cm the field speed was determined.

The spatiotemporal resolution of the Delta4, defined as the ability to accurately measure a moving field over time, was assessed using a sliding window experiment in which the projected positions of the MLCs were measured, and the projected field speed at isocenter was derived. A 22 × 2 ${\mathrm{cm}}^{2}$ field aperture at gantry angle 90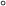 moved along the y‐axis between −5 cm and +5 cm (see, Figure 1c) at a constant dose rate and speed (see, Table 1). The speed of the field was defined by changing the amount of MU between control points at a set dose rate. Ten different speeds were evaluated without beam pausing, with six repetitions each, alternating between moving in the +*y* and −*y* directions (Figure 2a–c).

**TABLE 1:** The mean dose rate during measurement and intended field speed. $v_{intended}$ was calculated based on the actual dose rate during the measurement.

	MU									
	100	90	80	70	60	50	40	30	20	10
MU/min	412.9	410.2	410.2	410.8	410.7	410.7	410.6	410.6	409.9	410.5
$v_{intended}$ [cm/s]	0.7	0.8	0.9	1.0	1.1	1.4	1.7	2.3	3.4	6.8

Field speeds were determined by tracking dose over time (Figure 2a–c). The Delta4 *yz* dose data was simplified into a 1D *y* profile by taking at each *y*‐point, an average of all of the diode readings in the *z* direction. Field speeds of the Delta4 measurement were assessed between −2.5 cm and +2.5 cm in the *y*‐axis to use only the high‐resolution area of the detector board, minimizing effects due to under‐sampling. A smoothing spline curve was fitted through the 1D dose profile using the Matlab fit toolbox (version 2023a, The MathWorks Inc., Natick, Massachusetts), and maximum dose positions were obtained as a function of time. The field speed was determined as the slope of a linear fit applied through the field positions between −2.5 cm and +2.5 cm in the *y*‐axis. Comparison of the Delta4 and TRF field positions as a function of time was performed using correlation coefficients after down‐sampling the Delta4 dose data to 40 ms.

(1) $$v_{intended}=\frac{y_{MLC}\;\left(\mathrm{MU}/\min\right)}{MU_{beam}}$$

The intended field speeds (Equation 1) were calculated based on the MU to be delivered, the projected MLC travel distance ($y_{MLC}=10\;\mathrm{cm}$ at isocenter), and dose rate (MU/min) obtained from the TRF. Measured field speeds by the Delta4 were compared to TRF‐derived field speeds.

### 2.3 Demonstrator measurements

#### 2.3.1 Field position as function of time

Time‐resolved dose of the sliding window experiment was measured by the Delta4 and compared to the calculated time‐resolved 3D dose based on the TRF. The same measurement as performed in the Section 2.2.3 was used. Specifically, analysis was performed at the central detector row (in blue in Figure 2) and the time‐resolved dose readouts were compared.

#### 2.3.2 Time‐resolved plan QA of gated deliveries

The evolution of dose during a gated IMRT treatment was measured using the Delta4. A trigger signal based on a patient's free‐breathing respiratory motion in the cranial‐caudal direction (${\bar{A}}_{\mathrm{peak}-\mathrm{to}-\mathrm{peak}}$ = 18 mm, $\bar{T}$ = 5 s, $\bar{\mathrm{drift}}$ = 0.01 mm/min) was used, with an end‐exhale gating window of approximately 50% duty cycle. A clinical treatment plan for a pancreas patient was utilized, consisting of 11 beams with gantry angles: 200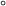, 220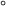, 275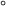, 350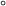, 25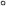, 50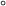, 75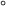, 85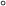, 148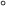, 160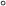, and 180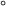. The total plan has 49 segments, with a prescribed dose of 8.0 Gy per fraction and a planned target volume of 85.0 ${\mathrm{cm}}^{3}$. Measurements were performed with and without beam gating. The TPS dose used for comparison was calculated with a 1 mm dose grid spacing and 0.5% statistical uncertainty per control point to reduce the influence of sampling artifacts and statistical uncertainty. Furthermore, the time‐resolved dose during the gated delivery was calculated based on the TRF and compared to the gated measurement.

Dosimetric analyses were performed for the first delivered segment of the treatment plan and the total cumulative dose. In the analysis of the first segment, the main focus was on dose accumulation in the high‐dose region. Therefore, the measurement points used for evaluation were within the $90^{\mathrm{th}}$ percentile of the first segment's maximum measured reference dose. The analysis performed on the total cumulative dose was performed using the measurement points within the $20^{\mathrm{th}}$ percentile of the maximum measured reference dose.

Additionally, a 3D global gamma analysis was performed on the gated and reference measurements relative to the planned dose.^21^ A criteria of 3%/3mm was used with a 20% lower dose threshold to the planned dose.

## 3 RESULTS

### 3.1 Validation experiments

#### 3.1.1 Reference measurements: Dose reproducibility and dose rate dependency

The short term reproducibility of the Delta4 expressed by the maximum dose deviation was 0.2% with respect to the mean measured dose. The dose rate dependency of the Delta4 showed a maximum dose deviation −0.3% during the 52 MU/min measurement with respect to the mean dose of the reference dose rate. At this dose rate, the time difference between the beam‐on time measured by the Delta4 and the TRF was 0.1 s (0.1%) during the 228.8 s measurement duration, as recorded by the Delta4. The dosimetric results are comparable to those found by de Vries et al., who found deviations of 0.2% and 1.0%, respectively.^16^

#### 3.1.2 Measurement uncertainty due to integration time

**FIGURE 3:**
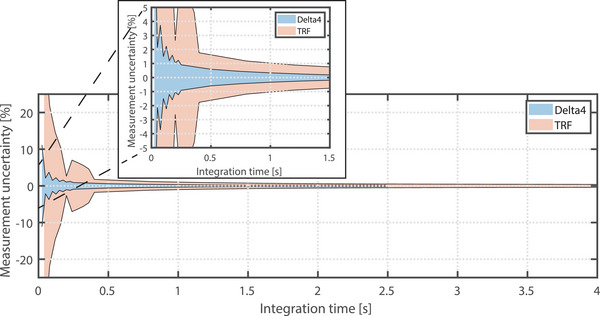
The temporal stability represented by the measurement uncertainty as a function of the integration time.

Figure 3 shows the measurement uncertainty as a function of the integration time for the Delta4 measured dose and TRF‐based dose calculations.

The measurement uncertainty becomes ≤ 1% after an integration time of 250 ms (10 samples) for the Delta4 measurement and 1200 ms (30 samples) for the TRF‐based dose calculation. For the comparisons of the measurements performed for Section 2.3.1, in which the temporal axis of the Delta4 was resampled based on the TRF's integration time, an integration window of 280 ms was used.

#### 3.1.3 Spatiotemporal resolution assessment

**FIGURE 4:**
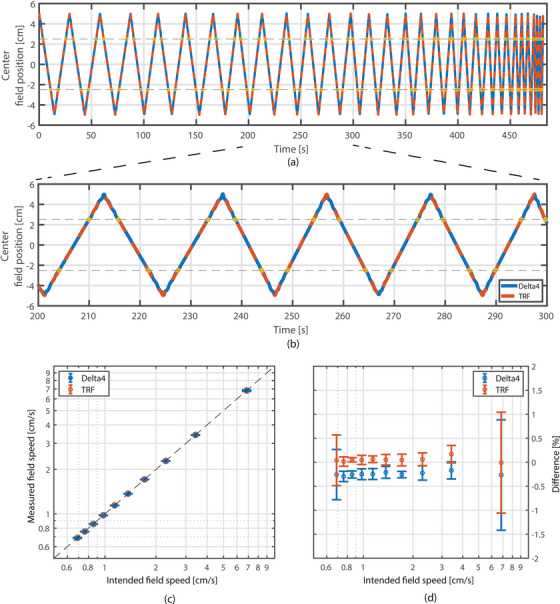
(a) Central field position over time determined by the Delta4 (blue) and TRF (red). The yellow markers show the intersection points with −2.5 and +2.5 cm limits. (b) Zoomed‐in region of (a). The sampling time of the Delta4 and TRF were 25 ms and 40 ms respectively. (c) Measured field speeds for Delta4 (blue) and TRF (red). The dashed black line represents the intended field speed. (d) The speed difference with respect to the intended field speeds for both the Delta4 (blue) and TRF (red). The error bars in (c) and (d) represent one SD on the measured field speed distribution. SD, standard deviations.

The center of the sliding window field for various field speeds and delivered without pausing the beam, is plotted in Figure 4a and b. The field positions determined from the Delta4 and TRF as a function of time were in good agreement with a correlation coefficient of almost unity. The yellow markers represent the ± 2.5 cm limits of the diode board between which the field speed was determined.

The Figure 4c shows the mean field speeds of the Delta4 and TRF versus the intended field speed with an error bar representing one SD of the measured field speed distribution, which were obtained by determining the slope between the yellow markers in Figure 4b. The relative field speed differences of the Delta4 and TRF relative to the intended field speed (as listed in Table 1) are shown in Figure 4d. The error bars represent one SD of the measured field speed distribution. The average difference between the Delta4 and the TRF field speeds was −0.3% ± 0.02% (1 SD). The average difference between the TRF field speeds and the defined $v_{intended}$ was 0.05% ± 0.05% (1 SD).

### 3.2 Demonstrator measurements

#### 3.2.1 Field position as function of time

**FIGURE 5:**
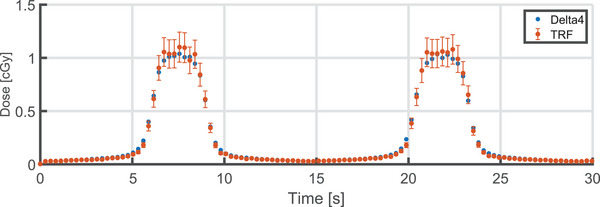
The averaged central *y* = 0 cm diode row dose readout in the sliding window experiment for the first 30 seconds. The dose readout was down‐sampled to 280 ms integration time. Error bars represent the 2 SD of uncertainty on the dose of the TRF‐based dose calculation. SD, standard deviations.

The mean dose for the central *y* = 0 cm diodes (see, Figure 2) during the first 30s (arbitrarily chosen) from the Delta4 (in blue) and TRF dose calculation (in red) is plotted in Figure 5 as a function of time, and agree well. The difference of the total cumulative dose between the measured and TRF‐based dose calculations was −0.8% ± 2.9% (2 SD).

#### 3.2.2 Time‐resolved plan QA of gated deliveries

**FIGURE 6:**
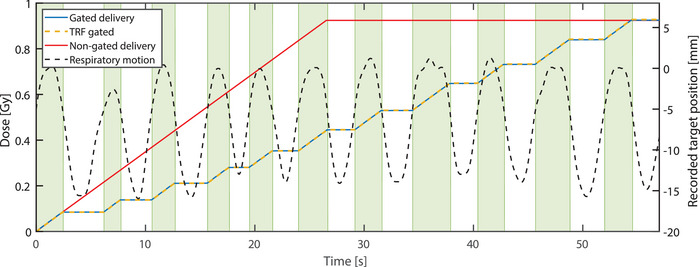
Accumulated dose as a function of time for the first segment of gated treatment delivery. The blue line represents the measured dose during gated treatment, while the red line shows the measured dose for the first segment of non‐gated delivery. The dashed yellow line indicates TRF‐based dose calculations. The dashed black line illustrates the respiratory trace used to trigger the beam. Green blocks indicate periods when the respiratory motion crosses the gating threshold, causing the beam to turn on.

Figure 6 illustrates the average measured cumulative dose of the 37 measurement points within the $90^{\mathrm{th}}$ percentile of the maximum measured dose of the first segment, as a function of time for the gated IMRT plan delivery. This shows the capability of the Delta4 to assess the delivered dose at any time point, and also to measure the beam on/off periods. The agreement between the gated and reference (non‐gated) measurement for the first segment was excellent with a difference of the cumulative dose of only 0.1% ± 0.1% (1 SD). The gated measurement and TRF‐based dose calculation also showed good agreement with a cumulative dose difference of −0.3% ± 0.7% (1 SD) for the first segment.

For the total cumulative dose on the measurement points within the $20^{\mathrm{th}}$ percentile of the maximum dose (316 measurement points), the Delta4 measurement matches well with the Monaco TPS dose, with differences of 1.8% ± 3.3% (1 SD) for gated and 1.7% ± 3.2% (1 SD) for non‐gated measurements, relative to the TPS dose. The gated TRF‐based dose calculations also showed excellent agreement to the gated measurement, with a difference of −0.3% ± 3.6% (1 SD).

Additionally, the 3D gamma analysis (3%/3mm) showed passing rates of 99.3% and 99.5% for the gated and reference measurements, respectively, relative to the TPS dose.

## 4 DISCUSSION

### 4.1 Validation experiments

#### 4.1.1 Reference measurements: Dose reproducibility and dose rate dependency

Short term dose consistency (0.2%) and dose rate consistency (−0.3%) performed with the time‐resolved software were consistent with the results found by the characterization study of de Vries et al.^16^ This shows that the time‐resolved research version of the Delta4 software is consistent with the clinical cumulative version.

#### 4.1.2 Measurement uncertainty due to integration time

To achieve a measurement uncertainty ≤ 1%, the Delta4 required 10 samples with an integration time of 250 ms (Figure 3). This indicates that the Delta4 exhibits higher measurement uncertainty during the start‐up phase or in short bursts of the beam (≤ 250 ms). A lower integration time can be used, but at the cost of greater uncertainty. If a higher accuracy is desired, a higher integration time can be used, or binning of multiple measurement points. However, as the dose delivery duration increases, the measurement accuracy also improves. Therefore, during typical measurements (e.g., plan QA), the measurement uncertainty should not be an issue as the duration of the delivery of a segment generally takes longer than 250 ms. Additionally, when investigating the cumulative dose, after 250 ms with a resolution of 25 ms, the uncertainty on the measurement is below 1%, and this will only decrease as the measurement duration increases. The TRF required a larger integration time to achieve a measurement uncertainty ≤ 1%. It should be noted that this is a recorded linac state, and not the actual dose output by the linac.

#### 4.1.3 Spatiotemporal resolution assessment

During this measurement it was shown that the Delta4 can be used to accurately measure with high temporal and spatial resolutions. The difference in the determined field speeds by the Delta4 and TRF, as shown in Figure 4c and d, was minor and is constant for each field speed. The error bars represent one SD of the measured field speed distribution and do not represent other uncertainties. Other uncertainties, such as the measurement uncertainty, may well contribute to the small systematic difference found, as the measurement of the different field speeds was performed in one continuous delivery. Also, the determination of the maximum dose position might be influenced by the fitting algorithm. Furthermore, the TRF field speeds showed excellent agreement to the $v_{intended}$.

### 4.2 Demonstrator measurements

#### 4.2.1 Field position as function of time

During the time‐resolved dose assessment of a sliding window, good correspondence with the TRF‐based dose calculations was observed, and fall all within the error bars, here representing 2 SD. This shows that the Delta4 can be used for measurement of the dose during a moving field. The differences observed between the Delta4 and TRF‐based dose calculations may well be introduced by using a research implementation for the dose calculations, which is in combination with the inherent temporal uncertainty of the TRF.

#### 4.2.2 Time‐resolved plan QA of gated deliveries

The gated plan QA measurement showed excellent correspondence with the references. These results demonstrate that the Delta4 can be used for measuring the time‐trace of the dose delivery of gated clinical plans. Furthermore, the time‐resolved, gated, TRF‐based dose calculations showed good agreement with the time‐resolved gated measurement.

### 4.3 Applications

Tools for QA of increasingly complex, dynamic dose deliveries, such as gating, MLC tracking and intra‐fraction adapted treatments, are required to enable clinical implementation. This paper demonstrates the suitability of the Delta4 system for gauging the time‐resolved dose of highly modulated treatment plans. The time‐resolved dose data provided by the Delta4 serve as a robust method for evaluating various linac parameters, such as field motion and delivered dose between control points. This is desired for investigating and validating new treatment modalities, beam modeling, or for finding the root cause of delivery errors. Furthermore, as shown here, the Delta4 can be used to assess novel applications that require high temporal and spatial resolution, such as time‐resolved dose calculations. Here we demonstrated a preliminary assessment of the TRF log file used for dose calculations, which yielded excellent agreement with the measured Delta4 dose. However, for commissioning purposes, tools like the Delta4 with independent time‐resolved dose measurement capabilities are needed.

## 5 CONCLUSION

The Delta4 with the time‐resolved dosimetry capabilities can measure radiation doses for static and dynamic dose deliveries.

## AUTHOR CONTRIBUTIONS

All authors participated and contributed to the data collection and this manuscript.

## CONFLICT OF INTEREST STATEMENT

The authors declare no conflicts of interest.
